# Imaging and Identification of Waterborne Parasites Using a Chip-Scale Microscope

**DOI:** 10.1371/journal.pone.0089712

**Published:** 2014-02-26

**Authors:** Seung Ah Lee, Jessey Erath, Guoan Zheng, Xiaoze Ou, Phil Willems, Daniel Eichinger, Ana Rodriguez, Changhuei Yang

**Affiliations:** 1 Department of Electrical Engineering, California Institute of Technology, Pasadena, California, United States of America; 2 Department of Microbiology, Division of Medical Parasitology, New York University School of Medicine, New York, New York, United States of America; 3 ePetri Inc., Pasadena, California, United States of America; 4 Department of Bioengineering, California Institute of Technology Pasadena, California, United States of America; Tufts University, United States of America

## Abstract

We demonstrate a compact portable imaging system for the detection of waterborne parasites in resource-limited settings. The previously demonstrated sub-pixel sweeping microscopy (SPSM) technique is a lens-less imaging scheme that can achieve high-resolution (<1 µm) bright-field imaging over a large field-of-view (5.7 mm×4.3 mm). A chip-scale microscope system, based on the SPSM technique, can be used for automated and high-throughput imaging of protozoan parasite cysts for the effective diagnosis of waterborne enteric parasite infection. We successfully imaged and identified three major types of enteric parasite cysts, Giardia, Cryptosporidium, and Entamoeba, which can be found in fecal samples from infected patients. We believe that this compact imaging system can serve well as a diagnostic device in challenging environments, such as rural settings or emergency outbreaks.

## Introduction

Waterborne protozoan parasite infections occur worldwide and pose a public health risk in both developed and developing countries [1], [2]. Outbreaks of these diseases are typically transmitted via the fecal-oral route in association with contamination of drinking water and food and poor sanitation at recreational water venues [2]. Symptoms typically involve severe diarrhea and fever, which can be life-threatening for young children, immune-deficient patients, and those living in the developing parts of the world1. In cases of outbreaks, rapid and accurate identification of the infection is important for the effective treatment and management of disease transmission. Among many species of protozoan parasites, *Giardia lamblia*, *Cryptosporidium parvum*, and *Entamoeba histolytica* are common etiological agents in most outbreaks.

The gold standard for the diagnosis of these parasitic infections is microscopic analysis of stool samples, complemented by polymerase chain reaction (PCR) or antigen detection technology for confirmation. Direct or concentrated stool smears are stained and examined under a microscope to identify the parasites in their cyst forms. Although simple and effective, imaging-based examination requires microscopes and trained microscopists on-site and the sensitivity and specificity of the tests are variable, depending on laboratory personnel expertise. Tests based on PCR or antigen detection techniques can be more reliable [3], but involve specialized laboratories (medium-sized clinical facilities) with advanced equipment, and thus are unsuitable for field settings. For point-of-care diagnostics, rapid immunoassay kits have been developed, but multiplexed assay is only available for two types of parasites (Giardia and Cryptosporidium [4]). In rural settings or emergency outbreak scenarios, imaging-based tests can still be a reliable and cost-effective solution for rapid diagnosis, provided that the imaging device and the screening process can be miniaturized and simplified.

Recognizing this need, low-cost chip-scale microscopes have been developed over the last few years, with reliable performance and compact configurations suitable for field-diagnostic applications [5], [6], [7], [8], [9]. Our recent development, named the sub-pixel sweeping microscopy (SPSM) [6], has shown high-resolution (660 nm) and wide-field (5.7 mm×4.3 mm) imaging with a low-cost and compact geometry. In the SPSM, a moving illumination source creates multiple shadow images of a sample placed directly on an image sensor. These low-resolution shadow images are then processed with the pixel super-resolution algorithm to provide high-resolution images. We have previously constructed a low-cost imaging system consisting of a complementary metal-oxide semiconductor (CMOS) image sensor, a smart phone screen as an illumination source, and a few Lego® blocks [6]. This imaging method is distinct from other lens-less microscopy methods [5], [10] in that it is fully capable of working well with samples that are connected contiguously. This point is a significant advantage because the SPSM can work well without the need for diluting the samples. Also, compared to fluorescence on-chip microscopy based on antibody staining [9], our bright-field imaging offers cheaper and simpler sample preparation with the expense of specificity, which may suit the field-applications.

The SPSM was originally designed to serve as a self-imaging Petri dish platform (ePetri). Here, we show that its general and simple-to-use microscopy imaging capabilities can be used more broadly. Specifically, in this study, we show imaging and identification of waterborne parasites with the SPSM imaging system using a light emitting diode (LED) array illumination. We demonstrate color imaging capability using a three-channel RGB LED array for identification of stained parasites. The three types of parasite cysts – Entamoeba, Giardia, and Cryptosporidium – are imaged and identified using the system. We further developed an automatic image reconstruction and screening method that can potentially be applied to digitalized diagnostic tests.

## Methods

**Figure 1:**
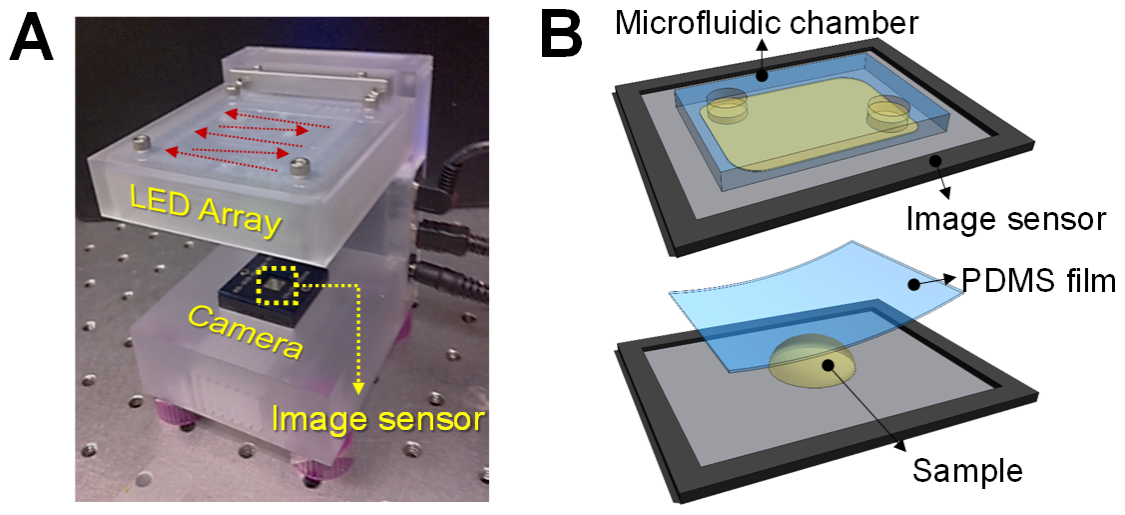
(a) Lens-less SPSM imaging system prototype. (b) A CMOS image sensor with a microfluidic chamber mounted on the sensor surface for sample loading. (c) A sample slide can be made directly on the image sensor.

Figure 1 shows the imaging system used in the experiment. The prototype SPSM system consists of a CMOS camera, an 8×8 RGB LED array and a control board. For imaging, we load the sample directly on the CMOS image sensor so that the light transmitted through the sample can be collected at the photodetector of each pixel in the image sensor. We used two schemes for sample loading. In one, microfluidic chambers were designed to contain a set volume of liquid sample and to hold the target object at the sensor's surface. The specimen could simply be pipetted into the chamber and allowed to settle for 5 min before imaging. In the other scheme, we made a wet film of the sample by placing a drop of liquid on the sensor and covering it with a cover glass or a transparent film. With both methods, the image sensor is reusable after flushing the channel and/or washing the sensor chip. Considering the low-cost of the semiconductor image sensors, the device can also be disposable.

**Figure 2:**
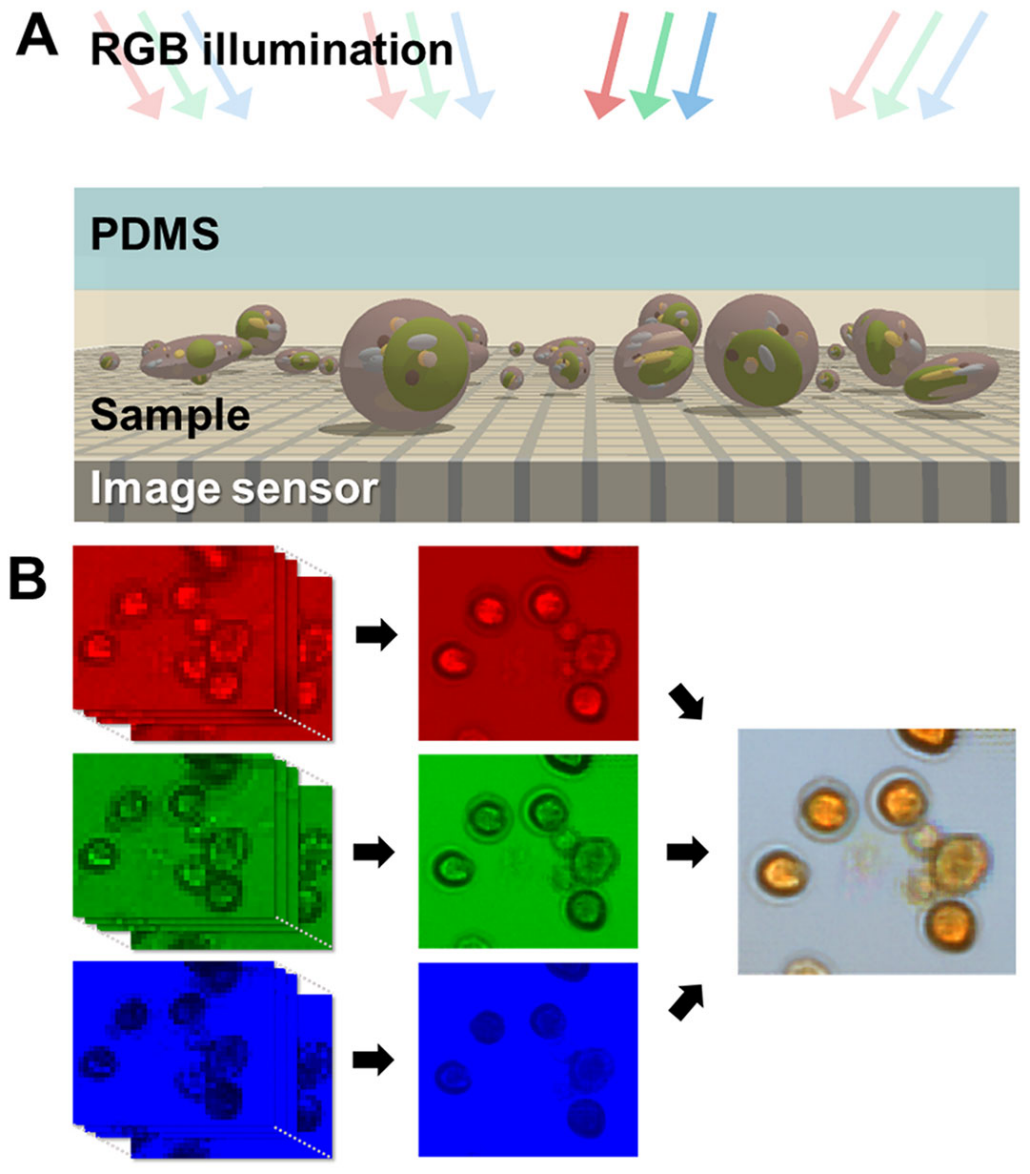
Schematic of the working principle of SPSM color imaging. (a) While the target objects rest on the surface of the image sensor, we sequentially turn on each LED in the RGB LED array illumination above and take sequences of low-resolution images. (b) Each low-resolution sequence is reconstructed into three monochromatic high-resolution images using the pixel super-resolution algorithm. The red, green, and blue channels are then combined into a single color image.

Once the sample is loaded onto the sensor, we sequentially turn on each LED in the 8×8 LED array. As the illumination scans, the image sensor captures the light transmitted through the sample under each LED. The raw images have low resolution (limited by the pixel size of the image sensor) but each frame represents a view of the object that is laterally shifted with respect to the next due to the varying angle of incidence in the scanning illumination. The pixel super-resolution reconstruction accounts for the different spatial information in the each of 64 low resolution images and restores a single high-resolution image (8× reduction in the pixel dimensions). For color imaging, we use three color LED illuminations to obtain red, green, and blue channels and then combine the images into a color image (Fig. 2). We use custom software that controls the LED array and the camera for the image capture. Once the data is acquired, the user can reconstruct high-resolution images of any area of interest with the software. The image acquisition and reconstruction process is similar to our previous demonstration of SPSM technique – except for the use of LED array as the light source – and the details of the technique are elaborated in ref. 6.

We used three types of parasite cysts for imaging. *G. lamblia* and *E. invadens* cysts were obtained from in vitro cultures of trophozoites [11], [12]. *E. invadens* is used as a model for *E. histolytica* because it can be induced to form cysts *in vitro* [13]. *C. parvum* oocysts were purchased from Excelsior Sentinel, Inc. Cysts were stained in suspension in Lugol's iodine (0.005%) or methylene blue (4.4 µM) before use.

Our optimized SPSM system used Aptina MT9P031 image sensors (2592×1944 pixels, pixel size 2.2 µm) as its sensing substrates. We removed the cover glass and the microlens array on each sensor surface (oxygen plasma cleaning, 120 W, 10 min) for direct access to the sensor pixels. Polydimethylsiloxane (PDMS) microfluidic channels were fabricated via a standard soft-lithography procedure and bonded onto the image sensor after oxygen plasma treatment (40 W, 30 s). Image sensors were reused after cleaning with deionized water in an ultrasonic bath for 30 s.

## Results and Discussion

**Figure 3:**
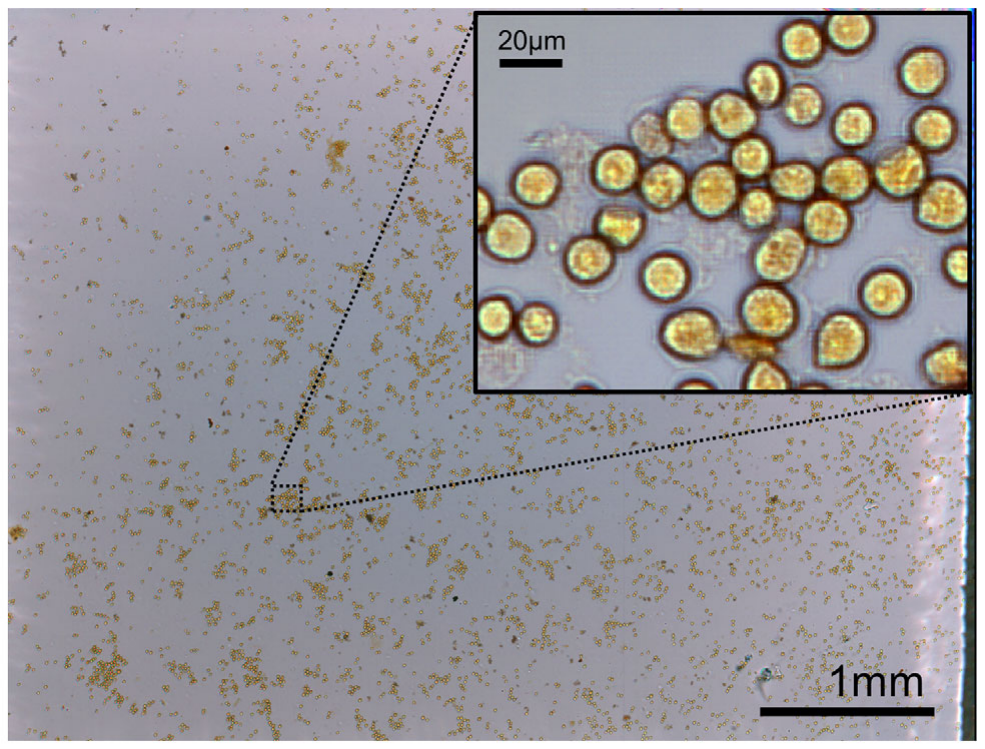
Full field-of-view (5.7 mm×4.3 mm) of *E. invadens* cysts with iodine staining. The inset shows a part of the reconstructed image.

Figure 3 shows a full-field image taken with our chip-scale microscope. A wet film of the *E. invadens* cysts was made directly on an image sensor by the method illustrated in Figure 1c. Previously, we have experimentally demonstrated 0.66 µm resolution with the SPSM technique (with 13×13 scanning and 2.2 µm pixels) [6], comparable to conventional microscope images taken using a 20× objective lens with a numerical aperture (NA) of 0.4. However, larger number of scanning directly relates to the increased image acquisition and reconstruction time and larger file size. We chose 8×8 scanning as a middle point between the trade-off between the resolution and imaging throughput. With 8×8 scanning, we expect that the resolution will be limited to three high-resolution pixels, which is 0.83 µm. The imaging area in the sensor measures 25 mm^2^, whereas the FOV of a 20× objective lens (Olympus, Plan N, NA 0.4) measures approximately 1 mm^2^. The data acquisition time is 2 min for 192 frames (64 frames for each color) and the raw sequence of the full FOV measures 930 megabytes.

Due to the large file size, the reconstruction of the full FOV takes a few minutes using a personal computer with an Intel i3 processor. However, the full-field high-resolution images are too large (20736×15552 pixels) to display on one screen. Instead, the user can zoom to a smaller area of interest, and the image can be reconstructed within a few seconds. Additionally, we implemented an automatic image reconstruction software, which detects all particles in the FOV from the raw image and returns reconstructed images cropped around each particle. The automatic detection and reconstruction further improves the reconstruction speed and can be streamlined with the image screening process for diagnosis.

**Figure 4:**
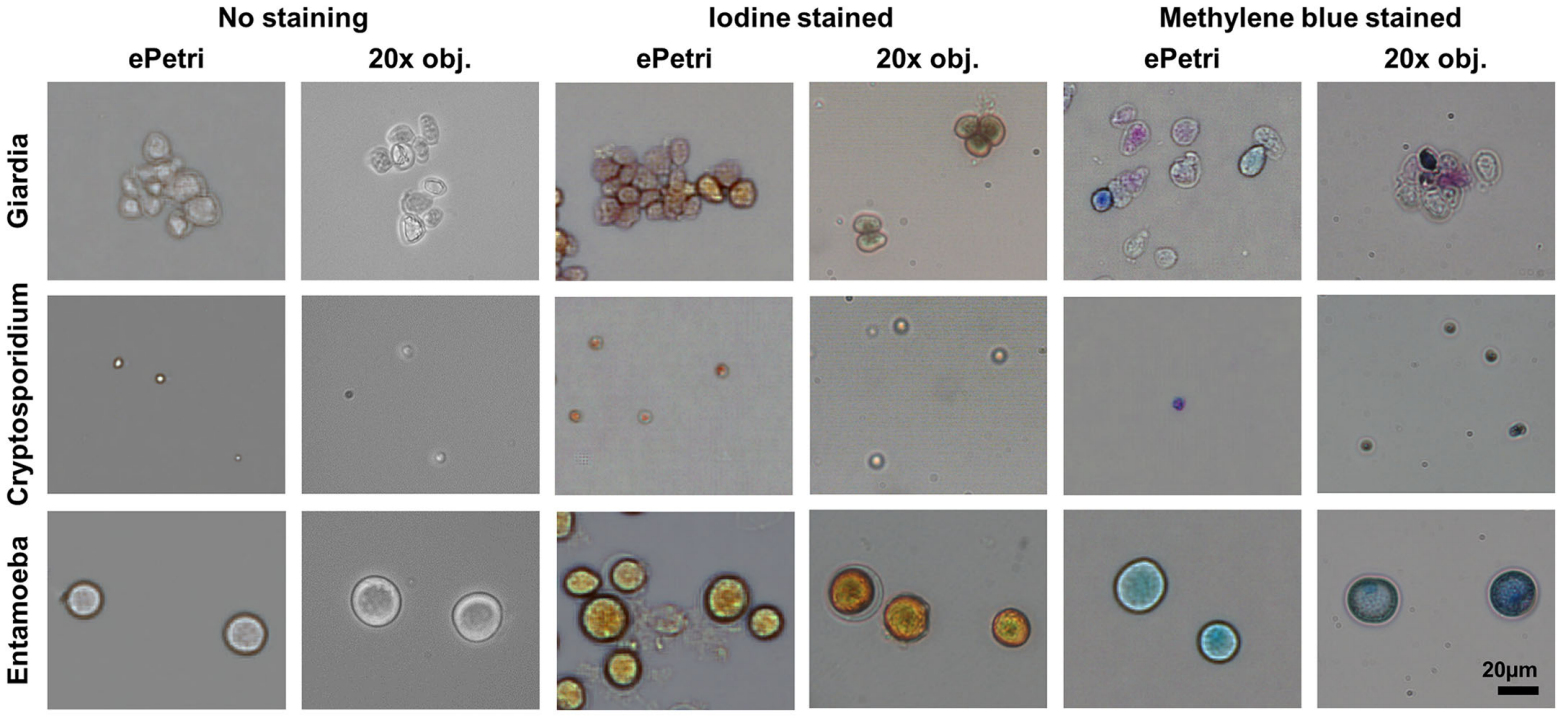
SPSM and 20× objective microscope images of Giardia, Cryptosporidium, and Entamoeba cysts. We imaged unstained cysts, iodine-stained, and methylene blue-stained cysts.

To demonstrate the imaging capabilities, we imaged three species of enteric parasite cysts with different types of staining (Fig. 4). Entamoeba, Cryptosporidium, and Giardia can be found in the stool of infected patients in cyst form, and the recovery of these cysts is a crucial part of the diagnosis. Iodine and methylene blue staining are widely used for the distinction of these cysts from other particles and debris in the fecal smear. In Giardia, different degrees of encystation are observed and differentiated by the varying intensity of staining, which is clearly observed in the SPSM images. The comparison with a 20× objective lens microscope images shows that our SPSM images provide enough resolution and color for the identification of these parasite cysts, especially with appropriate staining.

Our chip-scale microscope is advantageous for imaging a large number and a wide variety of target samples. Unlike digital-holography based techniques, the SPSM technique does not impose constraints on the target object, such as confluency or scattering properties [6]. With the SPSM, confluent cells and high-density smears can be imaged without the loss of resolution. Thus, we can screen more cells within the fixed imaging area. Additionally, the sample preparation steps and the resulting images of the SPSM technique are similar to conventional microscopy. This indicates that the experimental protocol and the diagnostic standards of conventional microscopy-based tests can be applied to our chip-scale microscopy technique without considerable modifications.

**Figure 5:**
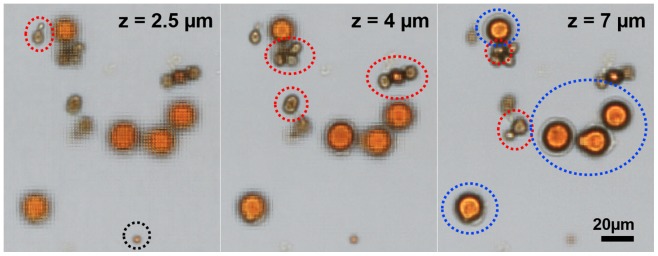
Digitally refocused images of parasite cysts. Entamoeba (blue), giardia (red), and cryptosporidium (black) can be found in different z-planes.

The SPSM method allows for digital refocusing of the images [14]. Each LED in the array illuminates the target object at a specific angle; thus, the sub-pixel shift between each frame varies with the vertical location of the target object. In the pixel super-resolution reconstruction process, we can arrange the low resolution frames with the specific shift corresponding to the depth of the imaging plane. As a result, the same set of raw data can reconstruct images at multiple depths and the objects with variable dimensions can be imaged without tuning the focus during image acquisition. Figure 5 shows images of Entamoeba, Giardia and Cryptosporidium cysts on the same chip, where the variations in the cyst sizes (5–20 µm) cause the cells to be placed in different imaging planes. The images can be digitally refocused at different heights, by using the simple linear relationship between the sub-pixel shift and the imaging depth [14] (z = 0 at the active layer of the image sensor). To speed up the reconstruction process, we only allow integer multiples of high-resolution pixel size for the sub-pixel shifts (i.e., the unit of sub-pixel shift is the pixel size of the sensor divided by the number of scanning in one direction), which gives us the z-step size of 1.5 µm.

It is worth noting that imaging thick samples causes cross-hatch artifacts in the image due to the shadows from the different height planes moving at different sub-pixel shifts upon sweeping of the illumination angle. These artifacts are periodic due to the periodic nature of the SPSM technique and can be suppressed by removing the corresponding spatial frequency components in the Fourier domain. However, complete filtering of these artifacts may result in the loss of resolution in the final image. Also, as the samples move further away from the sensor, the imaging resolution will deteriorate due to the diffraction of the shadows.

To verify the diagnostic potential of the SPSM technique, we performed a blinded experiment with images of Entamoeba, Giardia, and Cryptosporidium cysts. In the experiment, we asked two experienced researchers to identify the parasite species from randomized images. The researchers were given 60 randomized images of each cell type, taken with both SPSM and conventional microscopy (Fig. S1). In total, 360 images were analyzed. The samples used in the experiment were from separate batches of purified culture and the samples were stained with iodine. From this experiment, we were able to establish the identification accuracy of manual counting with false-positive and false-negative rates (Table 1). The accuracy of the identification was comparable between conventional microscopy and SPSM images for all three parasite species. This result indicates that images taken with the SPSM are suitable for distinguishing between the three parasites. The relatively low accuracy for Giardia in both SPSM and conventional images can be attributed to the variable sizes and shapes in the Giardia cyst sample and the errors were all false negatives, resulting in the false negative rate of 5% and 8.3% for SPSM and conventional images, respectively.

**Table 1:** Results of cell-type identification tests with conventional and SPSM images.

Sample	Method	Accuracy(%)	False Positive Rate (%)	False Negative Rate (%)
*Crypto-sporidium*	Conventional (manual)	100	0	0
	SPSM (manual)	100	0	0
	SPSM (automatic)	98	2.5	1.7
*Giardia*	Conventional (manual)	97	0	8.3
	SPSM (manual)	98	0	5
	SPSM (automatic)	96	2.5	6.7
*Entamoeba*	Conventional (manual)	100	0	0
	SPSM (manual)	100	0	0
	SPSM (automatic)	98	0.8	3.3

Our positive results prompted us to investigate the possibility of further streamlining the identification process, using an image-based automatic identification algorithm written in-house with the MATLAB image processing toolbox. We first detect the particles in the images by thresholding the images with the color and brightness variations. Next, we used blob analysis function to extract the area, diameter, aspect ratio, and convexity of the particles, which are used to identify the types of cysts. We optimized the algorithm by training it on a set of known SPSM cyst images (500 of each parasite species for a total of 1500 samples). We then tested its accuracy on the same set of images as was used in the manual experiment described above. The algorithm's accuracy and error rates are listed in Table 1. The algorithm performed slightly worse than manual counting but was, nevertheless, able to achieve a high level of accuracy. The errors were mostly attributable to clumps of cysts and irregularities. With the accuracy achieved, we believe that our automated identification can be applicable to the initial screening of parasite cysts in stool smear images, which can later be confirmed by the trained human as a means to expedite diagnosis.

The experimental format here is limited in scope in that it verified that the SPSM was able to distinguish between the parasites if they were the only objects present in the sample. To tackle parasite identification in a stool smear sample, the envisioned system would have to be able to distinguish parasites apart from generic particulates. While we expect image analysis would allow such a distinction to be made, the far larger proportion of generic particulates would likely negatively impact on the accuracy of such an analysis, even if the false-positive identification rate is low. We believe that one viable approach to this challenge would be to use fluorescently tagged antibodies that would preferentially attach to the parasites to help screen out generic particulates. We note that a small proportion of the generic particulates may also be fluorescently tagged, and thus lead to false-positive identifications. However, the combination of both bright-field image and fluorescence tagging should lead to a greatly suppressed false-positive rate.

## Conclusions

In summary, we have demonstrated on-chip color imaging of waterborne protozoan parasite cysts with the SPSM technique using LED array illumination. The proposed system achieves ultra-wide FOV (25 mm^2^) and high imaging resolution (<1 µm) with color capability. We showed that the system is capable of imaging three major types of water-borne parasite cysts, Entamoeba, Giardia, and Cryptosporidium, and the resulting images are suitable for both manual and automatic identification of the cyst types. We believe that, with further development, our chip-scale microscope can potentially provide a low-cost and portable solution to microscopy-based diagnosis of waterborne parasite infection in resource-poor settings.

## Supporting Information

Figure S1 **20× objective microscope (Left) and SPSM (Right) images of Cryptosporidium (top), Entamoeba (center) and Giardia cysts (bottom) used for the blind experiment and the automatic cell identification experiments.**
(JPG)
