# Supplementary figures and images for: Imaging and Identification of Waterborne Parasites Using a Chip-Scale Microscope
Source: PLoS One. 2014 Feb 26;9(2):e89712. doi: 10.1371/journal.pone.0089712 (PMC3935895; doi:10.1371/journal.pone.0089712)

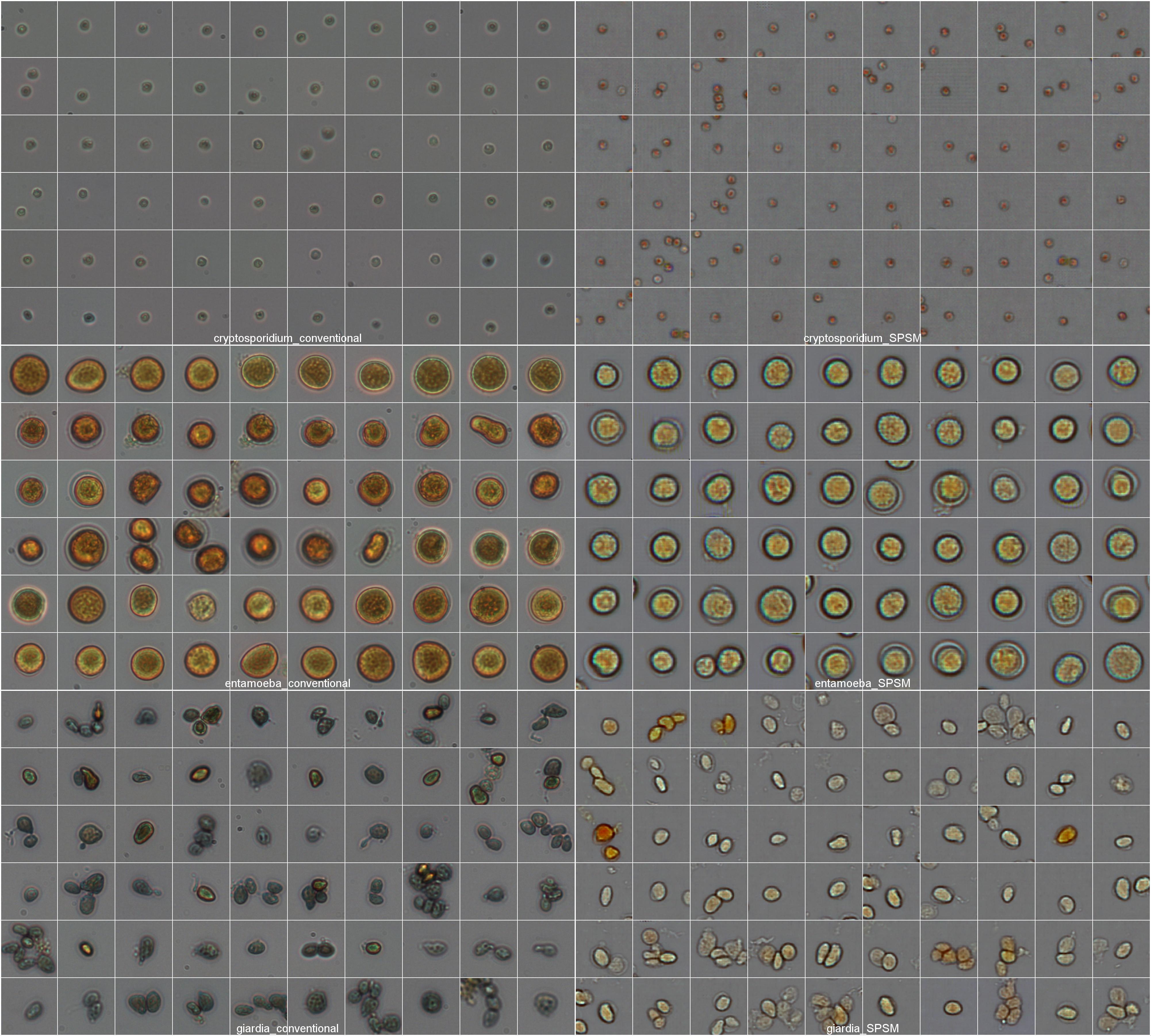

Supplement: Figure S1 — 20× objective microscope (Left) and SPSM (Right) images of Cryptosporidium (top), Entamoeba (center) and Giardia cysts (bottom) used for the blind experiment and the automatic cell identification experiments. (JPG) [file pone.0089712.s001.jpg]
